# Toltrazuril-Loaded Polymeric Nanocapsules as a Promising Approach for the Preventive Control of Coccidiosis in Poultry

**DOI:** 10.3390/pharmaceutics14020392

**Published:** 2022-02-10

**Authors:** Lana Flávia Baron, Francisco Noé da Fonseca, Shaiana Salete Maciag, Franciana Aparecida Volpato Bellaver, Adriana Mércia Guaratini Ibeli, Marcos Antônio Zanella Mores, Gabryelle Furtado de Almeida, Silvia Stanisçuaski Guterres, Ana Paula Almeida Bastos, Karina Paese

**Affiliations:** 1 Programa de Pós-Graduação em Ciências Farmacêuticas, Faculdade de Farmácia, Universidade Federal do Rio Grande do Sul, Av. Ipiranga, 2752, Porto Alegre 90610-000, RS, Brazil; lanaflaviabaron@hotmail.com (L.F.B.); gabryelle.almeida@gmail.com (G.F.d.A.); silvia.guterres@ufrgs.br (S.S.G.); 2 Embrapa Suínos e Aves, BR 153 Km 110 s/n, Concórdia 89715-899, SC, Brazil; adriana.ibelli@embrapa.br (A.M.G.I.); marcos.mores@embrapa.br (M.A.Z.M.); ana.bastos@embrapa.br (A.P.A.B.); 3 Campus CEDETEG, Universidade Estadual do Centro-Oeste do Paraná, Al. Elio Antonio Dalla Vecchia, 838, Guarapuava 85040-167, PR, Brazil; shaianamaciag@gmail.com; 4 Instituto Federal Catarinense, Campus Concórdia, Rd. SC 283 s/n, Concórdia 89703-720, SC, Brazil; franciana.bellaver@gmail.com

**Keywords:** Avian coccidiosis, *Eimeria* spp., toltrazuril, polymeric nanocapsules, Eudragit^®^ S100, poly-ε-caprolactone

## Abstract

Coccidiosis is a disease caused by intracellular protozoan parasites of the genus *Eimeria* that affect the intestinal tract of poultry. However, strain resistance and drug residue in the carcass have drawn the attention of the productive sector. The nanotechnology can improve the biological effect of drugs, reducing of administered doses and toxic effects. Due to this, toltrazuril-load polymeric nanoparticles based on Eudragit^®^ S100 (NCt) or poly-ε-caprolactone (LNCt) were developed to prevent coccidiosis in broilers. Nanoformulations were produced and showed homogeneous particle diameter distribution in the nanometer range (*z*-average and D (4.3) < 200 nm), negative zeta potential (<−8.93 mV), drug content ~100%, and encapsulation efficiency >90%. Cell viability assays using avian fibroblasts showed that LNCt presented no relevant toxicity up to 72 h. LNCt was then prophylactically administrated to chicken followed by challenge with *Eimeria* oocysts. The evaluation of the small intestine and cecum showed that the treatment with LNCt (3.5 mg/kg/day) in drinking water reduced the lesion scores and oocysts excretion, similar to the reference medicine containing toltrazuril (Baycox^®^, 7 mg/kg/day). The current study shows the potential protective use of nanoencapsulating anticoccidial drugs as a promising approach for the control of coccidiosis in poultry.

## 1. Introduction

Coccidiosis is a disease caused by intracellular protozoan parasites belonging to the genus *Eimeria* that affect the intestinal tract of animals. In poultry, *Eimeria* spp. (*E. tenella, E. mitis*, *E. maxima*, *E. acervulina*, *E. brunetti*, *E. necatrix,* and *E. praecox)* infections are responsible for important economic losses, as they reduce the zootechnical performance of the flock and, in the most serious cases, lead to the death of the birds [1]. Several risk factors are involved in cases of coccidiosis, such as the high density in aviaries and inadequate management of birds [2]. As it is a parasite that is widely distributed in the environment, several strategies are used for its control, such as the use of anticoccidial agents and vaccines, with the first approach being the most widespread. Usually, the drugs are incorporated into the feed or water and are supplied throughout the production cycle of the broilers. The problem with this strategy, however, is the emergence of drug-resistant isolates of *Eimeria* spp. [1]. Additionally, veterinary drug residues in poultry products can be transmitted to humans through the consumption of contaminated meat. Low and continuous exposure to antimicrobials triggers several pathological implications that are considered important public health problems. Chicken meat contaminated with drug residues can lead to antibiotic-resistant microorganisms, allergic manifestations, or changes in the microflora of the digestive tract of human beings [3]. These factors have drawn the attention of the productive sector, prompting the search for new approaches in management of this parasitosis.

In recent years, nanotechnology applied to veterinary products has become an emergent area of research and technological development. Different nanosystems have been investigated for animal therapeutics, breeding, reproduction, nutrition, etc. [4]. Drug nanoencapsulation is a promising alternative to conventional treatments because it is possible to improve the biological effect of drugs, reducing the number of administered doses and possible toxic effects [5].

Regarding coccidiosis, there are few reports in the literature on the use of nanostructured systems as a therapeutic strategy against infection by *Eimeria* spp. Some studies have particularly focused on inorganic nanomaterials, such as zinc [6] or silver [7]; however, their potential was limited due to lower efficacy compared with usual therapeutic agents, and carcass residue, which would face regulatory issues for use, respectively. Conversely, polymeric nanoparticles are interesting for this purpose since the materials used can be biodegradable and biocompatible, and the physicochemical properties can be modulated according to the final use [8]. Therefore, considering that Brazil is one of the world leaders in the production of broilers and the worldwide trend of reduction/withdrawal of the use of antimicrobial agents in animal production, nanoencapsulation of anticoccidial drugs has become a promising alternative to manage this parasitosis, adding value to the poultry production chain.

In view of the economic aspects of coccidiosis and animal welfare, this research aimed to develop polymeric nanocapsules containing toltrazuril (a wide spectrum anticoccidial drug) for the prophylaxis of *Eimeria* spp. infection in broilers, so that the drug release could be controlled and the biological response to the treatment could be improved, leading, consequently, to a reduction in the effective dose.

## 2. Materials and Methods

### 2.1. Chemicals

Toltrazuril was purchased from ChemCruz (Santa Cruz, CA, USA). Poly (ε-caprolactone) (PCL, Mw 14 kDa) and sorbitan monostearate were purchased from Sigma-Aldrich (Steinheim, Germany). Eudragit^®^ S100 was obtained from Evonik (Darmstadt, Germany). Polysorbate 80, ethanol, and acetone were purchased from Vetec (Rio de Janeiro, Brazil), and Capric/caprylic triglyceride (CCT) was obtained from Delaware (Porto Alegre, Brazil). Cell culture media and supplements were obtained from BD Difco™ (Franklin Lakes, NJ, USA). All other reagents and solvents used were of analytical or pharmaceutical grade.

### 2.2. Preparation of the Toltrazuril-Loaded Nanocapsules and Lipid-Core Nanocapsules

The polymeric nanocapsules were prepared using the interfacial deposition method of preformed polymer as described elsewhere with modifications [9]. Briefly, an organic phase containing the polymer (PCL or Eudragit^®^ S100, 200 mg), acetone (48 mL), ethanol (6 mL), oil (CCT, 320 µL), sorbitan monostearate (76 mg), and toltrazuril (10 mg) was injected into 53 mL of aqueous phase containing polysorbate 80 (154 mg), under magnetic stirring. The suspension was concentrated to 10 mL under reduced pressure so that the final concentration of toltrazuril was 1.00 mg/mL. Formulations prepared with PCL and Eudragit^®^ S100 were named LNCt and NC_S100_t, respectively. Blank formulations (without drug) were also prepared and named LNC and NC_S100_. All formulations were prepared in triplicate of batches, and after stored under refrigeration (8 °C).

### 2.3. Physicochemical Characterization

The particle sizes and the size distributions were determined by laser diffraction (*n* = 3) (Mastersizer 2000, Malvern Instruments, Worcestershire, UK). The diameter values were expressed by the volume-weighted mean diameter (d [4, 3]) and by the diameter at percentiles 10 (d(0.1)), 50 (d(0.5)), and 90 (d(0.9)) of the cumulative size distribution curve by volume (v) and by number (n) of particles. The volume-based values were used to calculate Span (Equation (1)).

(1) $$\mathrm{Span}=\frac{d_{0.9-d_{0.1}}}{d_{0.5}}$$

Additionally, the mean hydrodynamic diameter of the particles (*z*-average) and the polydispersity index (PDI) were determined using dynamic light scattering (DLS) (ZetaSizer Nano ZS, Malvern, UK). Each sample was diluted 500× in filtered ultrapure water, and all samples were analyzed in triplicate batches.

Zeta potential was measured by electrophoretic light scattering (ZetaSizer Nano ZS, Malvern, UK). Samples were diluted 500× in filtered NaCl 10 mM. All measurements were conducted at 25 °C in triplicate.

For pH, the formulations were evaluated (*n* = 3) without dilution at 25 °C using a calibrated potentiometer (UB-10, Denver Instruments, New York, NY, USA). Furthermore, the morphology of the formulations was analyzed by transmission electron microscopy (TEM, Jeol 1200 Exll, Tokyo, Japan) at 80 kV. The samples were diluted 1:10 (*v/v*) in filtered ultrapure water and deposited on copper grids (400 mesh) coated with formvar/carbon (Electron Microscopy Sciences. Hatfield, PA, USA). Staining was achieved with the *uranyl acetate* (UA) solution (2%, *w/v*).

Toltrazuril content in the nanocapsules was determined by liquid chromatography using a previously validated method [10]. The chromatographic system (Shimadzu Corporation, Kyoto, Japan) presented a C18 column (Phenomenex, 150 × 4.60 mm, 5 µm), which was eluted with an isocratic mobile phase (acetonitrile: water (80:20, *v/v*) with 0.1% acetic acid) at a flow rate of 1.0 mL/min and drug detection at 250 nm. Encapsulation efficiency (EE%) was determined based on the difference between total and free drug concentration. Free drug was quantified after ultrafiltration-centrifugation (1884× *g* for 30 min) in a Amicon^®^ Ultra centrifugal filter device (10,000 Mw, Merck Millipore^®^, Carrigtwohill, IRL). Quantifications were carried out using a standard curve of toltrazuril previously established (r > 0.99).

### 2.4. Evaluation of the Presence of Nanocrystals

The presence of nanocrystals was evaluated by complementary techniques. Initially, the analyses by nanoparticle tracking (NTA) were performed (NanoSight LM10, Nanosight, Amesbury, UK), where the formulations were diluted (10,000×) in 0.5 mL of ultrapure water and injected into the sample chamber cell. Images were recorded in sextuplicate for 10 s, at room temperature, using shutter and manual gain adjustments. Data were collected and analyzed using the device software (NTA 2.0 Build 127, Amesbury, UK) to identify and track the light individually scattered by the nanoparticles under Brownian motion.

Toltrazuril content of the formulations was determined under different storage conditions. For this purpose, the formulations were aliquoted in amber glass vials (two for each condition) and kept at room temperature (20 °C) or under refrigeration (8 °C). At different time intervals (1, 7, 15, 30, and 60 days), samples were collected based on two different procedures: one vial was kept at rest while the other was homogenized before pipetting. Afterwards, samples were quantified by HPLC, as previously described.

### 2.5. Evaluation of the Stability of the Formulations Exposed to Simulated Gastrointestinal Fluid

This procedure was adapted from the Boisen and Fernández method, which consisted in incubating the formulations in media simulating the avian gastrointestinal fluid [11]. First, the samples (2 mL) were added to the gastric fluid (35 mL) simulating the proventricle/gizzard one (0.1 M phosphate buffer, 0.2 M HCl, pH 2.0, and 25 mg/mL pepsin) for 30 min at 37 °C under magnetic stirring. Second, the intestinal fluid simulating the duodenum (15 mL; 0.2 M phosphate buffer, 0.6 M sodium hydroxide, pH 6.8, and 100 mg/mL pancreatin) was added to the reservoir and allowed to stand for 2 h. Finally, at the end of each step (30 min and 2 h), an aliquot was withdrawn for toltrazuril quantification and particle diameter analysis by HPLC and DLS, respectively.

### 2.6. Cell Viability Assays

The cytotoxic activity of LNCt, NC_S100_t, and free toltrazuril was evaluated using immortalized fibroblasts chicken line (CEC-32, BCRJ cat#0064). Cells were cultured in 96-well plates (5 × 10^5^ cells/well) in RPMI 1640 complete culture medium containing 10% fetal calf serum (FCS) in the presence of the nanoformulations, and incubated (37 °C, 5% CO_2_) for 24, 48, and 72 h.

The rate of cell membrane damage at different concentrations (35.25, 70.50, and 141.00 µM) was determined through the LIVE/DEAD^®^ Viability/Cytotoxicity Kit (Invitrogen^TM^, Eugene, OR, USA). To determine the rate of apoptotic cells after incubation with different concentrations of the nanoformulations (14.69, 29.38, 58.75, 117.5, 235, and 470 µM), cells were analyzed using APO-DIRECT™ Kit (Invitrogen^TM^, Carlsbad, CA, USA). All procedures were carried out according to the manufacturer instructions. In addition, to verify the effect of the nanocarrier itself, cells were incubated with blank formulations (LNC and NC_S100_) at equivalent volumes of the drug-loaded nanoformulation. In both assays, cells were analyzed using a flow cytometer (BD Accuri^TM^ C6 plus, BD Biosciences, San Jose, CA, USA) after the exposure time (24, 48, and 72 h) to determine the percentage of intact cell membranes and the percentage of live cells based on the fluorescence readings (10,000 events) according to the kit instructions.

### 2.7. Birds, Coccidial Infection, and Sampling

This study was approved by the Ethical Committee for Animal Experimentation of Embrapa Suínos e Aves (protocol N. 002/2018).

Broiler chicks (COBB line, 1-day-old, both sexes, 40–45 g) were weighed and divided into 7 groups (10 birds/group) and allocated to ensure similar initial weight in each group. They were housed in electrically heated battery cages; at 1st week the temperature was 35 ± 1 °C, and then was gradually decreased to 28 ± 1 °C in the 3rd week. The lighting schedule was 22 h light–2 h dark throughout the experiment. Feed and water were provided ad libitum. Chicks were inspected daily for any health problems, and mortality was recorded as it occurred. The groups were as follows: (G1) drinking water (DW) (negative control); (G2) DW + challenge (positive control); (G3) DW + Baycox® (7 mg/Kg/day) additive + challenge; (G4) DW + LNCt (7 mg/Kg/day) additive + challenge; (G5) DW + LNCt (3.5 mg/Kg/day) additive + challenge; (G6) DW + LNCt (1.75 mg/Kg/day) additive + challenge; (G7) DW + LNC additive + challenge.

At 13 and 14 day of age, birds were administered different doses of LNCt (G4, G5, and G6) in DW, as well as the blank formulation (G7-LNC, equivalent to the volume of the highest dose of LNCt) and Baycox^®^ (G3—commercial drug, 7 mg/kg/day). Control groups (G1 and G2) received only DW.

The challenge consisted of the administration of high doses of *Eimeria* vaccine to experimentally induce moderate intestinal damage and immune response [12]. Thus, at 16 day of age, all birds in the challenged groups (G2–G7) were gavaged with coccidial vaccine at 20× dose (Fortegra^®^, MSD Health Animal, Omaha, NE, USA), which contained live oocysts of *Eimeria mivati*, *Eimeria maxima*, *Eimeria acervulina*, and *Eimeria tenella*. The negative control (G1) was subjected to gavage with water.

Birds were weighed at 1, 13, 16, and 21 day of age, and feed consumption was determined at the end of the experiment (21st day). Excreta were collected at day 12 (the day before the treatments) and 17–21 (during 5 day after coccidial vaccine challenge for the challenged groups), which were stored in the freezer for oocyst counting and PCR analysis. Birds were euthanized at 21 day of age, and additional analysis was performed (intestinal gross lesion), and tissue samples from intestine were collected for evaluating duodenal, jejunal, and ileal morphology.

### 2.8. Oocyst Counting (EPG) and Lesion Score

Oocyst counting was performed by dilution and counts via microscope using a McMaster counting chamber (JA Whitlock & Co., Eastwood, NSW, Australia). Fresh feces (100 g) from each cage were collected and homogenized in 50 mL water. Homogenates of excreta/cage (6 g) were passed and stirred through a sieve with 60 mL saturated NaCl solution. A sample of the mixture was transferred into the two chambers of a McMaster slide (FEC source). After 5 min, the oocysts were counted under a microscope at 10× magnification. The number of oocysts was expressed as oocyst per gram of feces.

In addition, gross lesions (at 21 day of age) were scored visually according to the method of Johnson and Reid by personnel blinded to treatment based on lesion scores ranging from 0 to 4, where 0 corresponds to the normal status with no gross lesions, 1 to small scattered petechiae, 2 to numerous petechiae, 3 to extensive hemorrhage and mucosal injury, and 4 to extensive hemorrhage that gives a dark color to the cecal intestine [13]. Furthermore, for the assessment of the gross lesion, four species present in the live vaccine target the duodenal loop and upper jejunum (*E. acervulina*), mid-intestinal area (*E. maxima*), and the cecum (*E. tenella*) were used according to the criteria of Conway and McKenzie [14].

### 2.9. DNA Extraction

Fecal samples were collected from each pen at 12 day to evaluate the presence/absence of *Eimeria* spp. at the beginning of the experiment. The DNA was extracted using the Fast Stool DNA mini kit (Qiagen, Hilden, Germany) following the manufacturer’s instructions. A DNA extraction positive control of excreta containing 3 *Eimeria* spp. (*E. maxima, E. tenella,* and *E. acervulina)* was also included in each extraction batch. DNA samples were quantified in a Biodrop spectrophotometer (Biodrop, Cambrigde, UK).

At 21 day of the experiment, the DNA of the 56 cecal content (8 samples/group) was extracted using the DNA Stool mini kit (Qiagen, Hilden, Germany) following the manufacturer’s instructions. As previously described, a DNA extraction positive control of excreta containing 3 *Eimeria* spp. was also included in each extraction batch.

### 2.10. Identification of Eimeria spp. Using qPCR

To perform the *Eimeria* spp. identification, primers and probes for *E. acervulina* [15], *E. tenella* [15], *E. maxima* [15], and *E.* spp. [16] were used for real-time PCR assays (Supplementary Materials Table S1). The qPCR reactions were performed in Quantstudio 6 (Applied Biosystems, San Francisco, CA, USA) real-time PCR equipment in a 15 μL containing 2× GoTaq Probe qPCR Master Mix with ROX as passive reference dye (Promega, Madison, WI, USA), 0.13 μM of each primer, 0.09 μM of probe, and 1.5 μL of DNA. The cycling conditions used were 95 °C for 2 min, followed by 40 cycles at 95 °C for 15 s, and 60 °C for 60 s. Samples were run in duplicate, and negative and positive controls for *E. maxima, E. tenella,* and *E. acervulina* were added in each reaction. Standard curves were performed using a Gblock DNA fragment (IDT, Coralville, IA, USA) containing the sequences for the 4 assays (*E. maxima*, *E. tenella*, *E. acervulina*, *E.* spp.) to be evaluated in this study. A 10-fold dilution (10^6^−10^1^ copies/μL) was performed and run in each qPCR reaction. PCR efficiency and absolute quantification were based on 10^(−1/slope)^. After the qPCR reactions, threshold cycles (Ct) and mean quantities (copies/PCR) were collected and submitted to statistical analysis. To verify the differences among the groups, the Kruskal–Wallis followed by the post hoc Dunn’s test was applied using R. A *p*-value < 0.05 was considered significant.

### 2.11. Intestinal Morphological Analysis

Fragments of small intestine (duodenum, jejunum, and ileum) and cecum were also collected at 21 day of age for histological analysis. Tissue samples were fixed in 4% (*v/v*) buffered formaldehyde, dehydrated, cleared, and embedded in paraffin. Two serial sections/bird (3 μm thick) were stained with Hematoxylin and Eosin for analysis. The lesions were scored from 0 to 4, considering the parameters according to Table 1.

Two more serial sections/bird (3 μm thick) were stained with Alcian Blue pH 2.5 (American Forces Institute of Pathology, 1992; Lev and Spicer, 1964) and Fast red Stain Kit (Vector labs, Burlingame, CA, USA) following the manufacturer’s protocol. Slides after deparaffinization and rehydration were incubated in acetic acid 0.5 M for 3 min and then in Alcian Blue solution (10 g/L in acetic acid 0.5 M, pH 2.5). After washing in water, the slides were dehydrated and mounted. The number of Alcian Blue-positive cells along the villi was counted by light microscopy. After staining, treatment-blind histological analysis was performed on ten complete and well-oriented villi and ten crypts per section. Sections were assessed for villus height (VH), crypt depth (CD), goblet cells (GC) counts (per villus), and GC density (per μm of VH) by light microscopy (200× magnification) (Supplementary Materials Figure S1). Density of goblet cells was calculated as the number of goblet cells per unit of surface area (mm^2^).

### 2.12. Mucosal Sample Analysis

Mucosa samples from 10 bird/cage were scraped from 10 cm of the ileum (5 cm proximal to the Meckel’s diverticulum) at 21 day of age. Production of mucosal secretory IgA (E33-103, Bethyl Laboratories, Montgomery, TX, USA) and polymeric IgM receptor/secretory component (E33-102, Bethyl Laboratories, Montgomery, TX, USA) were determined by ELISA following the manufacturer’s protocol. The total protein in the mucosal homogenates was measured colorimetrically using a commercially available kit (Pierce BCA Protein Assay, Thermo Fisher Scientific, Rockford, IL, USA) with bovine serum albumin as standard. Collected explants were homogenized in ice-cold PBS (10%, *w/v*) to obtain the supernatant.

### 2.13. Statistical Analysis

Data are presented as mean ± standard deviation (SD) or standard error mean (SEM) for physicochemical and biological parameters, respectively. Parametric data were analyzed using ANOVA (post hoc Tukey’s test or Dunnett’s test) or as non-parametric data using Kruskal–Wallis (post hoc Dunn’s test). Differences were considered statistically significant at *p* < 0.05.

## 3. Results and Discussion

### 3.1. Physicochemical Characterization

All formulations (LNCt and NC_S100_t) were successfully prepared following the methodology proposed and consisted of a white opalescent liquid with bluish tint due to the Tyndall effect, indicating the presence of colloidal structures (nanometric particles). Regarding the *particle size distribution* profile, both NC_S100_t and LNCt presented homogeneous monomodal distribution in the nanometer range (data not shown).

**Figure 1:**
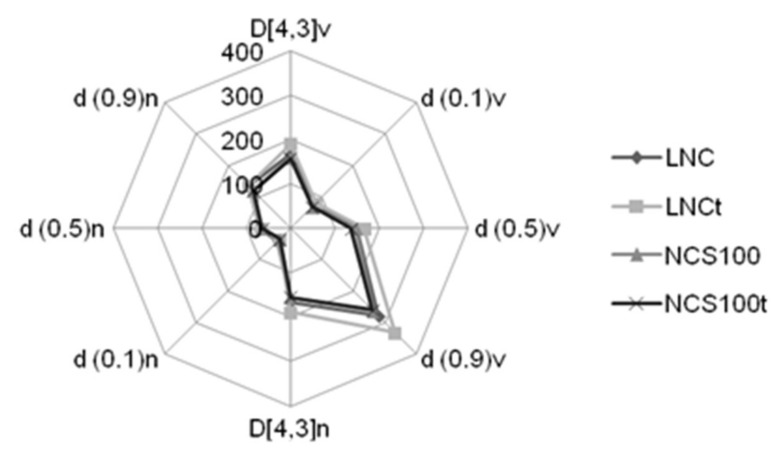
Fingerprint of nanoformulations containing toltrazuril obtained by laser diffraction. The values represent the average for three batches. LNC = blank lipid core nanocapsules; NCS100 = blank Eudragit^®^ S100 nanocapsules; LNCt = toltrazuril-loaded lipid core nanocapsules; NCS100t = toltrazuril-loaded Eudragit^®^ S100 nanocapsules formulations; D[4,3]v: volume-weighted mean diameter by volume of particles; d(0.1)v: diameter by volume at percentile 10 under the distribution curve; d(0.5)v: diameter by volume at percentile 50 under the distribution curve; d(0.9)v: diameter by volume at percentile 90 under the distribution curve; D[4,3]n: volume-weighted mean diameter by number of particles; d(0.1)n: diameter by number at percentile 10 under the distribution curve; d(0.5)n: diameter by number at percentile 50 under the distribution curve; d(0.9)n: diameter by number at percentile 90 under the distribution curve.

In Figure 1, the radar chart presents the fingerprint of the nanoformulations developed (containing or not toltrazuril) based on particle diameter distribution in terms of volume and number. Bianchin et al. [17] proposed this approach (fingerprint from laser diffraction analysis) to select promising drug delivery systems, where unimodal size distributions are advantageous over multimodal profiles, since drug release behavior and ability to cross biological barriers are dependent on the particle size [17]. In this sense, the *study found* that these *profiles* present the same identity and are compatible with the expected characteristics for nanosuspensions.

D[4,3] values for NC_S100_t and LNCt were 157 ± 2 and 190 ± 6 nm, respectively (Table 2). Regarding the dispersion of diameter values (span), all formulations developed had values ranging from 1.3 to 1.5, indicating homogeneity in the distribution of diameter values. For *z*-average and PDI values, the inclusion of the drug did not lead to a significant difference between blank or loaded formulation. Moreover, all formulations had negative zeta potential, being the values for the Eudragit^®^ S100 nanocapsules, in module, greater than the formulations obtained with PCL. In addition, the formulations presented pH ranging from 4 to 5, indicating their compatibility for oral use. Overall, the physicochemical parameters obtained are in accordance with other similar nanoformulations reported [5,18,19,20,21,22].

**Figure 2:**
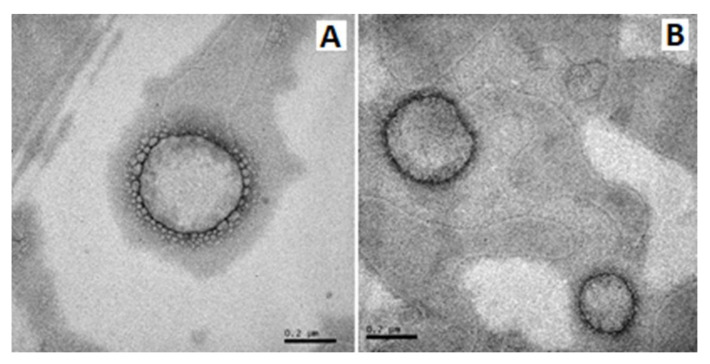
Photomicrographs obtained by transmission electron microscopy at 80 kV, with a magnification of 100,000×. (**A**) Toltrazuril-loaded lipid core nanocapsule (LNCt); (**B**) Toltrazuril-loaded Eudragit^®^ S100 nanocapsule (NC_S100_t). Bar equal to 0.2 µm.

The formulations had toltrazuril contents close to 100% and an encapsulation efficiency greater than 90% (Table 2), indicating that the drug concentration was near the theoretical volume (1 mg/mL) in both formulations. TEM analyses showed nanocapsules with spherical morphology (Figure 2); in addition, the nanometric diameter could be confirmed, corroborating the other values obtained through indirect measurements (laser diffraction and dynamic light scattering).

### 3.2. Presence of Nanocrystals

Many studies have shown the ability of PCL and Eudragit^®^ S100 to encapsulate drugs; however, their encapsulation efficiency may vary according to the inherent drug characteristics. Oliveira et al. evaluated the drug association mechanism in LNC and proposed an algorithm based on the distribution coefficient of the drug (logD) to suggest its distribution within the nanocarrier [23]. Briefly, model drugs with different LogD were nanoencapsulated, and the EE% was determined after sequential dilution (1:10, 1:100, and 1:1000); according to the correlation profile of EE% and LogD, the drug location within the nanocarrier pseudophases was proposed as mostly located in the oily core, in the polymeric wall, or in the external aqueous phase. Toltrazuril has a logD of 3.74 (pH 5.0) and could be classified as type V (drug supersaturation with nanocrystal formation in the external aqueous phase and no precipitation detected after preparation) or VI (lipophilic drugs mainly associated with the core of the nanocapsules). In this context, we evaluated the presence of nanocrystals to confirm the possible type for toltrazuril.

NTA analysis of NC_S100_ and NC_S100_t formulations indicated an overlap in the scattered light intensity points in the left quadrants of the graph, without the presence of scattered light points in the upper right quadrant of the graph (Supplementary Materials Figure S2A). Similarly, LNC and LNCt formulations presented no points distributed in the upper right quadrant (Supplementary Materials Figure S2B). According to Jornada et al., the light scattering intensity of colloids varies with the presence of drug nanocrystals in suspension, which leads to scattered points in the upper right quadrant related to their refringent nature [24]. Thus, based on the profiles found, the developed formulations did not contain nanocrystals in their composition in short term.

**Figure 3:**
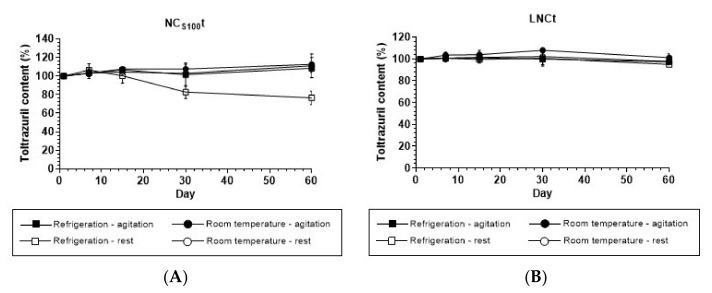
Toltrazuril content in (**A**) Eudragit^®^ S100 nanocapsules (NC_S100_t) and (**B**) lipid core nanocapsules (LNCt) submitted to room temperature (RT) and refrigeration as a function of time, with agitation or rest prior to pipetting for quantification. Values represent mean ± SD (*n* = 3).

Conversely, due to the nanosystem instability, the drug may precipitate under long-term storage. Then, regarding the drug content of the formulations stored for up to 60 days at room temperature and under refrigeration, the results indicate that there was no loss of content under most of the conditions studied, remaining nearly 100% (Figure 3). However, a decrease in toltrazuril content was observed in the NC_S100_t formulation submitted to refrigeration and rest; after 30 and 60 days, the content percentages were 80% and 74%, respectively. This decrease in toltrazuril content of NC_S100_t as a function of time may be associated with the formation of drug nanocrystals over time due to refrigeration. To confirm this hypothesis, the formulations stored at 8°C were observed under polarized light (Supplementary Materials Figure S3), and nanocrystals only occurred in the NC_S100_t formulation when stored under refrigeration.

### 3.3. Stability of the Formulations Exposed to Simulated Gastrointestinal Fluid

In general, no significant difference was observed in the physicochemical parameters for both formulations (NC_S100_t and LNCt) (Supplementary Materials Table S2). Considering the *z*-average, the values ranged from 207 to 211 nm for LNC, from 152 to 159 nm for NC_S100_, from 176 to 181 nm for LNCt, and from 151 to 154 nm for NC_S100_t. Additionally, simulated gastrointestinal fluid is a complex mixture containing salts, enzymes, and altered pH (both acidic and alkaline), which would affect the nanosystem homogeneity. However, no alteration was verified in terms of homogeneity since PDI values remained below 0.2 in all steps for all formulations. In contrast, when considering the drug content, only NC_S100_t presented a reduction in toltrazuril content of about 10%, indicating that LNCt has higher stability in gastrointestinal fluid.

### 3.4. Cytotoxicity Assay

**Figure 4:**
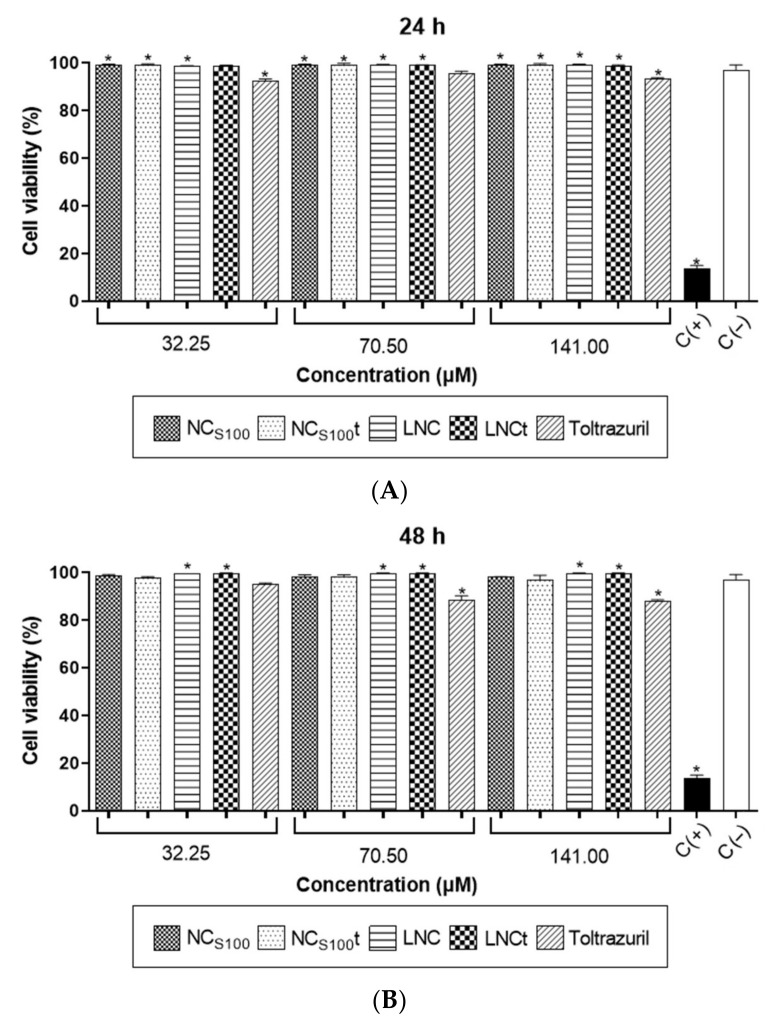

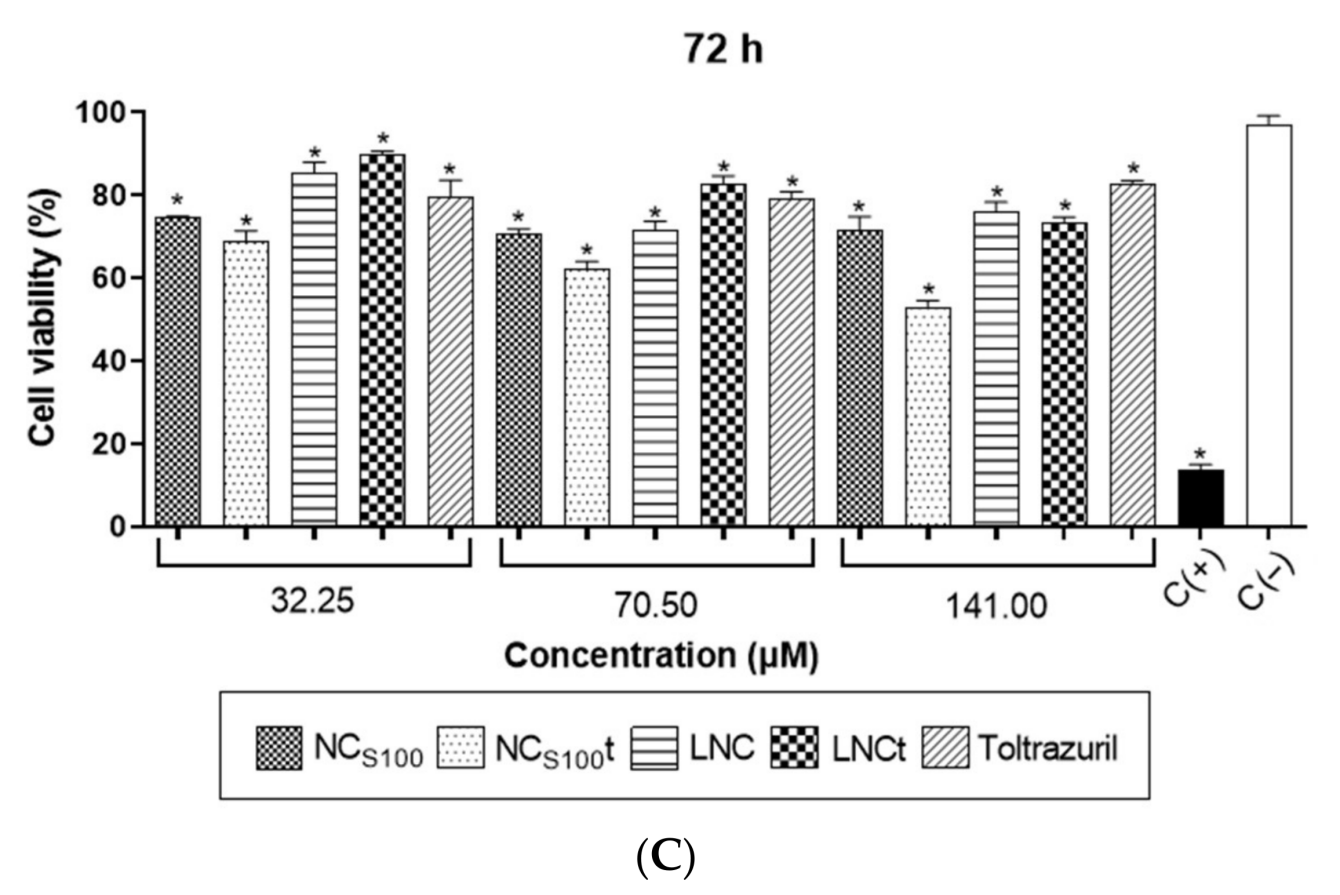
Cell viability of chicken fibroblasts (CEC-32 cell line) exposed to toltrazuril-loaded nanocapsules based on membrane damage (LIVE/DEAD^®^ Viability/Cytotoxicity Kit) after (**A**) 24, (**B**) 48 and (**C**) 72 h. Data represent mean ± SD (*n* = 3−4). * *p* < 0.05 vs. C(−) (one-way ANOVA, post hoc Dunnett’s test). NC_S100_ = blank Eudragit^®^ S100 nanocapsules; NC_S100_t = toltrazuril-loaded Eudragit^®^ S100 nanocapsules; LNC = blank lipid core nanocapsules; LNCt = toltrazuril-loaded lipid core nanocapsules; C(+) = positive control; C(−) = negative control. Blank formulations (NC_S100_ and LNC) were added the same volume, respectively, to the drug-loaded nanoformulation.

During the development of new drug delivery nanosystems, it is important to establish the safety of the proposed nanoformulations prior to the administration in animals, and cell viability assays, including different markers, are the most common techniques for this purpose. Considering that the nanoformulation is intended for administration in poultry, we carried out the experiments using the CEC-12 cell line, which consists of immortalized chicken fibroblasts. Figure 4 presents the effect of toltrazuril-loaded nanocapsules on CEC-12 cells after 24, 48, and 72 h. The incubation for 24 or 48 h did not damage cell membranes since all viability rates were above 80% independent of the concentration and the treatment. On the contrary, after 72 h of incubation, cell viability reduced significantly in comparison with non-treated cells (negative control) for all treatments. A notorious dose-dependent effect was observed only for NC_S100_t (69.00, 62.35, 53.20%) despite both the blank nanocarrier (NC_S100_) and toltrazuril in solution presenting cell viability above 70%. For LNC and LNCt, viability rate remained acceptable (>80%).

**Figure 5:**
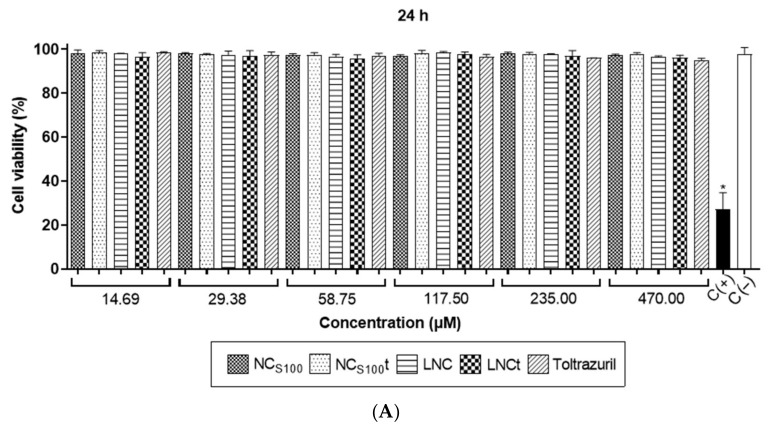

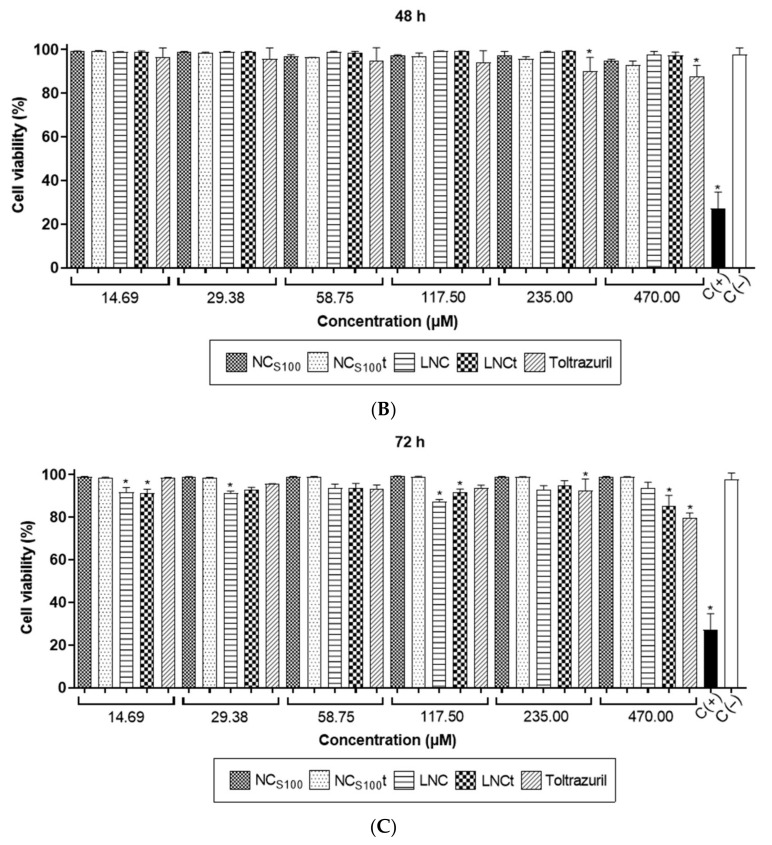
Cell viability of chicken fibroblasts (CEC-32 cell line) exposed to toltrazuril-loaded nanocapsules based on DNA damage (APO-DIRECT^®^ kit) after (**A**) 24, (**B**) 48 and (**C**) 72 h. Data represent mean ± SD (*n* = 3−4). * *p* < 0.05 vs. C(−) (one-way ANOVA, post hoc Dunnett’s test). NC_S100_ = blank Eudragit^®^ S100 nanocapsules; NC_S100_t = toltrazuril-loaded Eudragit^®^ S100 nanocapsules; LNC = blank lipid core nanocapsules; LNCt = toltrazuril-loaded lipid core nanocapsules; C(+) = positive control; C(−) = negative control. Blank formulations (NC_S100_ and LNC) were added the same volume, respectively, to the drug-loaded nanoformulation.

In addition, to verify the alterations in apoptosis mechanisms induced by the nanoformulations, we evaluated the rate of DNA damage (Figure 5) using a wider range of concentration (14.69–470.00 μM). No relevant alteration was observed for any of the treatments and the concentrations evaluated. Additionally, incubation time did not affect the cell viability in terms of apoptosis since all rates ranged above 80%, except for the positive control (<30%).

Many studies have reported the effects of polymeric nanocapsules on different cancer and normal cell lines, using different in vitro assays [25,26]. For LNC, Zanotto-Filho et al. observed that blank LNC did not reduce the viability of glioma cells up to 96 h of incubation; however, the incorporation of curcumin led to high rates of cell death [27]. A similar result was described by Sandri et al., where peripheral blood mononuclear cells and polymorphonuclear cells had no alteration in terms of viability after 24 h of treatment with blank LNC [5]. As expected, cells presented good tolerability to Eudragit^®^ S100 nanocapsules up to 48 h of incubation, in agreement with another study that evaluated indomethacin-loaded NC_S100_ in mouse fibroblasts (L929 cells) for 24 h, which resulted in cell viability up to 80% [28]. Moreover, the exposition of different cancer cell lines (HT-29 and HCT-116) to blank NC_S100_ for 24 and 48 h resulted in cell viability ranging from 60% to 100%; however, the nanoencapsulation of thymoquinone increased cell death [29]. In general, based on our findings, we concluded that both LNC and NC_S100_ are considered non-cytotoxic for chicken fibroblasts, but NC_S100_t tended to alter cell viability in terms of membrane damage (markedly at 72 h) in comparison with LNCt. Thus, we decided to choose LNCt to carry on the in vivo biological evaluation.

### 3.5. Dilution of LNCt in Water Prior to the Treatment of the Birds

Toltrazuril and LNCt were administered in the drinking water during 24 h. The formulation was diluted in three different concentrations to guarantee the dose for each treatment (G4: 7, G5: 3.5, and G6: 1.75 mg/kg/day). However, considering that nanocarriers can suffer instability due to the dilution process, such as drug displacement, the nanotechnological characteristics were assessed soon after dilution and after 24 h (Supplementary Materials Table S3). Drug contents in the treatments were 109.00%, 106.31%, and 103.71% relative to the theoretical values of the concentration for each group (G4, G5, and G6, respectively). The association efficiency of toltrazuril after dilution was above 99% for the three dilutions evaluated after preparation and 24 h later. Additionally, the particle diameter for each treatment was also evaluated, and no difference among the diameters before and after the dilutions was observed (~180 nm). These findings indicate that LNCt was stable after dilution and proper for use in drinking water.

### 3.6. OPG Analysis and Eimeria Identification Using qPCR

No oocyst was detected in excreta from chickens at 12 and 17 day for all groups. Oocysts were not detected in chickens non-challenged (G1) and treated (7 mg/kg/day) early with Baycox^®^ (G3) and LNCt (G4) at 18, 19, 20, and 21 day. Considering the *Eimeria* spp. DNA quantification in excreta, there was no detection for the non-challenged group (G1), while the detection for the other groups increased from day 19 to 21 (Supplementary Materials Figure S4). For the positive control group (G2), the analysis showed a higher number of oocysts in excreta at 18 (1.1 × 10^4^/g excreta), 19 (6.6 × 10^3^/g excreta), 20 (1.1 × 10^4^/g excreta), and 21 day (1.66 × 10^6^/g excreta). The proportion of oocyst number was similar between the positive control (G2) and the blank formulation (LNC, G7); the oocysts were detected in G7 at 19 (6.6 × 10^3^/g excreta), 20 (6.6 × 10^3^/g excreta), and 21 day (1.11 × 10^6^/g excreta). Regarding G5 and G6 (middle and low dose of LNCt), the oocysts were only detectable at day 21 (0.6 × 10^3^/g and 1.3 × 10^3^/g excreta, respectively), but oocyst excretion was lower compared with infected and non-treated groups (G2 and G7). For the excreta DNA quantification (Supplementary Materials Figure S5), the amount of log copy number/PCR was from 5.44 to 14.64 and it was possible to verify that the G3 and G4 groups present reduced *E.* spp. counting compared with G7 (adj *p*-value < 0.05). When the other groups were compared, no significant differences were observed.

### 3.7. Pathological and Histological Lesions Due to Coccidiosis

As presented in Table 3, the administration of the vaccine produced the specific lesions for all strains (lower extent for *E. tenella*) in the non-treated challenged birds (G2, positive control), and a similar result was found for LNC (G7). In contrast, the treatment with the commercial medicine (Baycox^®^, G3) resulted in no gross changes in the intestine. Despite that, the LNCt treatments (G4, G5 and G6) showed mild lesions.

**Figure 6:**
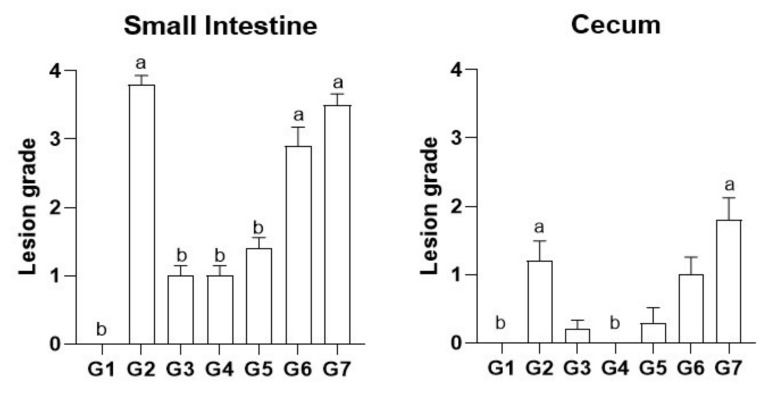
Histological scoring of *Eimeria* spp. lesions in chickens prophylactically treated with toltrazuril formulations. Data expressed as means ± SD (*n* = 9–10 per group); a *p* < 0.05 vs. G1 and b *p* < 0.05 vs. G2 (non-parametric Kruskal–Wallis test, post hoc Dunn’s test). G1 = control (−); G2 = control (+); G3 = Baycox^®^ (7 mg/kg/day); G4 = LNCt (7 mg/kg/day); G5 = LNCt (3.5 mg/kg/day); G6 = LNCt (1.75 mg/kg/day); G7 = LNC (volume equivalent to G4).

Histopathological analyses of small intestine (duodenum, jejune, and ilium) and cecum are presented in Figure 6. No obvious lesions for the small intestine and cecum were observed in G1 group (chicken unchallenged). On the contrary, bleeding (mild to moderate) in the small intestine and in cecum was detected in all challenged groups (G2–G7), indicating that the birds in the present study had mild-to-moderate/severe coccidial infection. Non-treated and challenged birds (G2) and birds treated with blank nanoformulation (G7) showed moderate to severe lesions (score 3–4). The administration of toltrazuril (G3–G6) reduced significantly (*p* < 0.05) the lesions in the small intestine compared with positive control; however, a mild reduction in lesion severity was observed for the lower dose of LNCt (G6). In cecum, the lesion severity was found to be heterogenous among groups, still the administration of toltrazuril (G3–G5) seemed to improve the lesions.

### 3.8. Histomorphometric Measurements of the Small Intestine

The histomorphometry of duodenum, jejunum, and ileum of the chickens at 21 days of age are presented in Table 4. For a healthy chicken, villus height/crypt depth (VH:CD) ratio is expected to be at least 5 (five parts of villus per one part of crypt) [30], as observed in G1. Epithelial sloughing was observed in non-treated challenged chickens (G2) and treated with blank formulation (LNC, G7). The challenge resulted in a significant decrease in the duodenal, jejunal, and ileal VH:CD ratio (*p* < 0.05) in comparison with non-challenged birds (G1). The administration of Baycox^®^ (G3) prior to the challenge led to a significant improvement in duodenal VH:CD ratio (*p* < 0.05) compared with G2 (positive control), as well as the treatment with LNCt (G4, G5) for both duodenal and jejunal VH:CD ratio (*p* < 0.05). Interestingly, no significant increase in the ratio was observed in ileum for G3 and G4, despite being numerically higher than positive control (G2). Overall, the treatment with toltrazuril-based formulations (G3–G5) enhanced the VH:CD ratio in duodenum and partially in jejunum. Goblet cells are responsible for secreting mucins that constitute the mucus layer, which acts as physical barrier and presents immunological functions to inhibit pathogens from entering epithelial cells [31].

**Figure 7:**
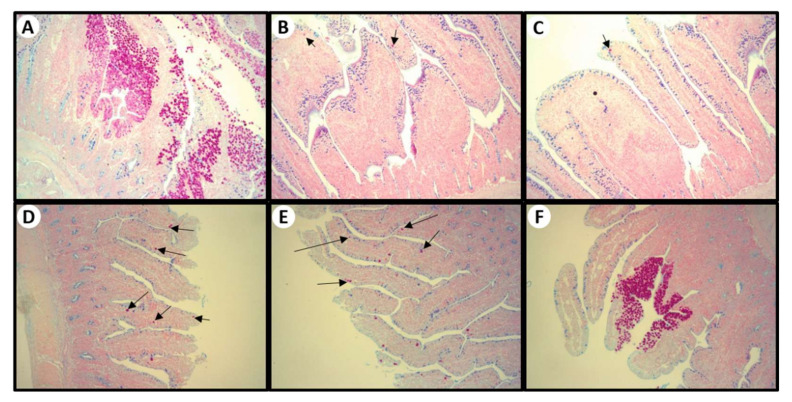
Small intestine stained with Hematoxylin and PAS. (**A**) positive control: intestine showing sloughing and desquamation of the intestinal lining and necrosis of the cells and different developmental stages of *Eimeria* spp. *Eimeria* oocysts are within the enterocytes of the columnar epithelium. (**B**) Baycox^®^ (7 mg/kg/day), (**C**) LNCt (7 mg/kg/day), (**D**) LNCt (3.5 mg/kg/day), and (**E**) (1.75 mg/kg/day): intestine showing mild degree of *Eimeria* spp. (arrows). (**F**) LNC (volume equivalent to LNCt 7 mg/kg/day): intestine showing severe contamination by *Eimeria* spp. Magnification, 100×.

Coccidial infection reduces the density of goblet cells in the intestine, affecting the bird development and performance. In the present study, the challenge dose reduced significantly (*p* < 0.05) about 50% of the goblet cells amount in the non-treated chickens (G2) in comparison with the non-challenged ones (G1) in all segments of the small intestine. This is *similar* to what *occurred* with the birds treated with LNC (G6), showing that the nanocarrier itself had no protective effect against *Eimeria* spp. infection. The early treatment with Baycox^®^ (G3) and LNCt (G4 and G5) prevented the reduction in goblet cells significantly (*p* < 0.05) when compared with positive control (G2), markedly in duodenum, with mild and minor effect on jejunum and ileum, respectively (Figure 7).

Regarding mucosal thickness, the results were inconclusive since no significant difference was found, even for the non-treated challenged group (G2) compared with the negative group (G1). For villus thickness, in all challenged groups (G2–G7), the villi thickened in comparison with G1, but the treatment with toltrazuril formulations (G3–G6) presented only a tendency to reduce villus thickness.

### 3.9. Mucosal Sample Analysis

In ileum, the concentration of IgM and IgA exhibited an increase in challenged groups compared with the negative group, except for the group treated with LNCt 7mg/kg (G4). Table 5 shows the course of IgM and IgA concentration in groups according to the treatment. Overall, the challenged groups (with or without treatment (G2–G7)) showed IgA and IgM contents higher than the unchallenged group (G1). Non-treated challenged birds presented significant (*p* < 0.05) IgA and IgM titers in comparison with the negative group (G1: 789.7 ± 198.3 and 987.0 ± 470.9 ng/mg protein for IgA and IgM, respectively; G2: 1268 ± 216.7 and 2141 ± 340.9 ng/mg protein for IgA and IgM, respectively). Regarding the treatment with toltrazuril, Baycox^®^ group (G2) resulted in values for IgA and IgM also significantly higher (*p* < 0.05) than the negative group (G1). Additionally, LNCt treatments (G4, G5 and G6) reduced significantly (*p* < 0.05) the IgM concentration in ileal mucosa compared with positive control (G2); G4 and G5 presented lower values than the group treated with Baycox^®^.

In this study, the main goal was to evaluate the potential of using nanoencapsulated toltrazuril for the prophylaxis of coccidiosis in broilers. The administration of nanocapsules (LNC and LNCt), as well as the commercial medicine (Baycox^®^), did not alter food and water intake, since no difference in weight development was observed in comparison with the negative control group (G1). Considering the oocysts counting, as expected no EPG counting was observed before challenge; on the other hand, *Eimeria* oocysts counts surged higher at 2 days after challenge in the non-treated challenged group (G2), which could explain the rapid acquisition of active immunity, such as IgA and IgM [32]. Moreover, toltrazuril-treated animals had negligible excretion of oocysts, which was confirmed by qPCR of *Eimeria* spp., being possible to detect *Eimeria* DNA in treated groups, although in low amounts. An important aspect to highlight is that there was no *Eimeria* amplification in G1 (negative control), evidencing that no cross contamination occurred in the experiment.

Regarding the mucosal analysis, the mucosal immune system in the gut faces the challenge of eliminating potential pathogens while maintaining a mutually beneficial relationship with the commensal microbiota. IgM is the predominant isotype produced after initial exposure to a novel antigen, while the secretory antibodies of the IgA class (SIgA) represent the first line of antigen-specific immune defense in the gut lumen. Although the SIgA has not yet been shown to have any protective action in chickens during coccidiosis [33], the challenge (coccidial vaccine overdose) can affect the immunological barrier function of intestinal mucosa. *Eimeria* infection stimulates the humoral immunity; therefore, the increased production of immunoglobulins (IgA and IgM) is nevertheless greater in the parasitized area (duodenum) [33,34,35], and antibody levels are related to the severity of the infection [36], as well as the level of exposure to the parasite [37]. Toltrazuril treatment in the infected groups (Baycox^®^ and LNCt) prevented or reduced *Eimeria* infection in the birds, as described in EPG and qPCR findings. They demonstrated that the infected-treated groups had a lower infection and, consequently, a lower production of immunoglobulins, when compared with the animals in the infected-untreated group (positive control).

Considering the efficacy of the prophylaxis of the infection caused by *Eimeria* spp. in terms of histopathological parameters, groups G4 and G5 (7 and 3.5 mg/kg/day, respectively) presented mild gross lesions, which were similar to G3 (Baycox^®^, 7 mg/kg/day). In addition, corroborating these results, the histological analysis in the small intestine and cecum indicates that the lesion scores were similar among groups. These findings suggest that the nanoencapsulation of toltrazuril made it possible to halve its dose for the treatment of coccidiosis, since the same response was observed in comparison with the reference medicine. In this context, the ability of nanoencapsulation to reduce drug dose was already evidenced in other studies, such as for PCL nanocapsules containing curcumin in the treatment of glioma in rats [27] and cloxacillin in the treatment of mastitis in sheep [38]. Moreover, to the best of our knowledge, this is the first report that evaluated polymeric nanocapsules containing an anticoccidial drug (toltrazuril), being a promising approach for the prophylactic control of *Eimeria* spp. infection in poultry.

## 4. Conclusions

Toltrazuril, a wide spectrum anticoccidial drug, was successfully nanoencapsulated in polymeric nanocapsules based on Eudragit^®^ S100 and PCL, which presented overall proper physicochemical properties and stability. The in vitro evaluation on chicken fibroblast showed that PCL nanocapsules did not alter cell viability up to 72 h, indicating its potential for the in vivo experiment. The prophylaxis of broilers with toltrazuril-loaded nanocapsules followed by challenging with oocysts of *Eimeria* spp. significantly reduced the lesion scores in the small intestine, as well as the excretion of oocysts. Furthermore, the administration of half of the dose of nanoencapsulated toltrazuril resulted in similar effects in comparison with the reference medicine. Thus, considering the relevance of coccidiosis for the poultry chain, nanoencapsulation of anticoccidial drugs is a promising approach for the preventive control of this disease, improving bird health and, consequently, animal production performance.

## Figures and Tables

**Table 1:** Lesions scores from *Eimeria* spp. infection for histological analysis.

Score	Small Intestine	Cecum
0	No mucosal forms of *Eimeria*	No mucosal forms of *Eimeria*
1	Rare or few forms of *Eimeria* in the mucosa	Few forms of *Eimeria* in the mucosa
2	Moderate amount of mucosal *Eimeria* forms in at least one of the fragments	Few foci with abundant forms of *Eimeria* in the mucosa
3	Large number of *Eimeria* forms in one of the fragments, associated with mucosal injury	Several foci with abundant forms of *Eimeria* in the mucosa
4	Large number of *Eimeria* forms in at least two of the fragments, associated with mucosal injury	Many foci with abundant forms of *Eimeria* in the mucosa

**Table 2:** Physicochemical characteristics of nanocapsules and lipid core nanocapsules containing toltrazuril or not.

	LNC	NC_S100_	LNCt	NC_S100_t
d[4,3] (nm)	173 ± 8	158 ± 4	190 ± 6	157 ± 2
Span	1.34 ± 0.27	1.40 ± 0.01	1.52 ± 0.04	1.41 ± 0.01
*z*-average (nm)	193 ± 5	164 ± 1	191 ± 6	166 ± 4
PDI	0.11 ± 0.02	0.12 ± 0.02	0.11 ± 0.02	0.10 ± 0.00
pH	5.08 ± 0.21	4.15 ± 0.21	5.14 ± 0.21	4.08 ± 0.37
Zeta potential (mV)	−8.93 ± 0.57	−12.73 ± 1.57	−7.72 ± 0.41	−11.53 ± 0.50
Content (%)	n.d.	n.d.	100.26 ± 1.13	98.86±2.12
Encapsulation efficiency (%)	n.d.	n.d.	93.25	92.68

Values represent mean ± SD (*n* = 3). LNC = blank lipid core nanocapsules; NC_S100_ = blank Eudragit^®^ S100 nanocapsules; LNCt = toltrazuril-loaded lipid core nanocapsules; NC_S100_t = toltrazuril-loaded Eudragit^®^ S100 nanocapsules formulations; n.d.: not determined.

**Table 3:** Scores of *Eimeria* species-specific lesions of chickens prophylactically treated with toltrazuril formulations.

Group	Strain		
	*E. acervulina*	*E. maxima*	*E. tenella*
G1	0 b	0 b	0
G2	3.70 ± 0.15 a	3.60 ± 0.22 a	0.7 ± 0.21
G3	0 b	0 b	0
G4	0 b	0.10 ± 0.10 b	0
G5	0 b	0.10 ± 0.10 b	0
G6	0.10 ± 0.10 b	0.40 ± 0.16 b	0.50 ± 0.22
G7	3.20 ± 0.20 a	3.30 ± 0.15 a	1.40 ± 0.22 a

Data expressed as mean ± SD (*n* = 9–10 per group). a *p* < 0.05 vs. control (−) and b *p* < 0.05 vs. control (+) (non-parametric Kruskal–Wallis test, post hoc Dunn’s test). G1 = control (−); G2 = control (+); G3 = Baycox^®^ (7 mg/kg/day); G4 = LNCt (7 mg/kg/day); G5 = LNCt (3.5 mg/kg/day); G6 = LNCt (1.75 mg/kg/day); G7 = LNC (volume equivalent to G4).

**Table 4:** Histomorphometric measurements of the small intestine in broilers prophylactically treated with toltrazuril formulations at 21 days of age.

Segment	Group	Goblet Cells (cells/mm^2^)	Mucosal Thickness (mm)	Villus Thickness (mm)	VH:CD Ratio
**Duodenum**	G1	123.50 ± 26.81 b	36.11 ± 4.10	121.30 ± 30.12 b	5.81 ± 0.80 b
	G2	63.25 ± 17.04 a	37.64 ± 5.43	213.40 ± 40.71 a	2.80 ± 0.46 a
	G3	109.70 ± 17.00 b	45.53 ± 12.36	164.60 ± 40.87 b	4.63 ± 0.69 a, b
	G4	122.60 ± 24.29 b	46.41 ± 16.76	175.60 ± 39.80 a	4.92 ± 0.87 a, b
	G5	119.10 ± 16.99 b	44.96 ± 8.44	186.40 ± 29.45 a	4.73 ± 0.71 a, b
	G6	93.19 ± 27.52 a, b	41.01 ± 6.32	177.10 ± 40.58 a	3.57 ± 0.72 a
	G7	69.34 ± 29.57 a	45.75 ± 5.48	183.90 ± 40.08 a	1.36 ± 0.30 a, b
		**Goblet cells (cells/mm** ^**2**^ **)**	**Mucosal thickness (mm)**	**Villus thickness (mm)**	**VH:CD ratio**
**Jejunum**	G1	131.50 ± 24.63 b	34.40 ± 3.81	108.00 ± 11.58 b	5.83 ± 0.79 b
	G2	70.26 ± 33.67 a	36.14 ± 3.18	177.10 ± 24.11 a	2.68 ± 0.67 a
	G3	104.60 ± 26.71	40.18 ± 5.50	140.50 ± 35.57	3.53 ± 0.73 a
	G4	107.00 ± 27.22 b	37.35 ± 4.26	161.10 ± 31.48 a	3.76 ± 0.84 a, b
	G5	90.50 ± 35.80 a	38.97 ± 6.27	145.30 ± 36.70	3.61 ± 0.79 a, b
	G6	72.67 ± 23.94 a	36.13 ± 4.78	153.80 ± 39.92 a	2.80 ± 0.58 a
	G7	84.58 ± 37.38 a	40.35 ± 5.78	156.20 ± 32.19 a	3.00 ± 0.98 a
		**Goblet cells (cells/mm** ^**2**^ **)**	**Mucosal thickness (mm)**	**Villus thickness (mm**)	**VH:CD ratio**
**Ileum**	G1	121.70 ± 15.47 b	35.08 ± 7.16	99.98 ± 18.46 b	5.52 ± 0.79 b
	G2	69.97 ± 27.02 a	35.71 ± 3.33	149.40 ± 24.50 a	2.92 ± 0.73 a
	G3	101.00 ± 33.07	41.01 ± 5.85	135.10 ± 26.31	3.45 ± 1.08 a
	G4	96.56 ± 39.99	38.18 ± 4.11	140.80 ± 19.05 a	3.20 ± 1.06 a
	G5	59.07 ± 27.51 a	38.94 ± 4.27	144.70 ± 50.61 a	2.69 ± 0.93 a
	G6	57.47 ± 18.07 a	37.35 ± 4.05	139.30 ± 33.81 a	2.43 ± 0.52 a
	G7	71.19 ± 25.09 a	39.04 ± 1.72	163.40 ± 36.37 a	2.86 ± 0.59 a

Data expressed as mean ± SD (*n* = 9–10 per group). a *p* < 0.05 vs. control (−) and b *p* < 0.05 vs. control (+) (ANOVA, post hoc Dunnett’s test). VH:CD = villus height/crypt depth; G1 = control (−); G2 = control (+); G3 = Baycox^®^ (7 mg/kg/day); G4 = LNCt (7 mg/kg/day); G5 = LNCt (3.5 mg/kg/day); G6 = LNCt (1.75 mg/kg/day); G7 = LNC (volume equivalent to G4).

**Table 5:** Immunoglobulin profile of broilers prophylactically treated with toltrazuril formulations at 21 days of age.

Group	IgA (ng/mg protein)	IgM (ng/mg protein)
G1	789.7 ± 198.3 b	987 ± 470.9 b
G2	1268 ± 216.7 a	2141 ± 340.9 a
G3	1210 ± 515.6 a	1520 ± 559.7 a, b
G4	968 ± 478.3	826.1 ± 194.5 b
G5	1034 ± 270.8	1048 ± 331 b
G6	1102 ± 282.5	1150 ± 486.9 b
G7	1075 ± 205.9	2059 ± 548.7 a

Data expressed as mean ± SD (*n* = 8–10 per group). a *p* < 0.05 vs. control (−) and b *p* < 0.05 vs. control (ANOVA, post hoc Dunnett’s test). G1 = control (−); G2 = control (+); G3 = Baycox^®^ (7 mg/kg/day); G4 = LNCt (7 mg/kg/day); G5 = LNCt (3.5 mg/kg/day); G6 = LNCt (1.75 mg/kg/day); G7 = LNC (volume equivalent to G4).

## Data Availability

The data generated or analyzed during this study are included in this published article and its Supplementary Information Files.

## Supplementary Materials

The following supporting information can be downloaded at: https://www.mdpi.com/article/10.3390/pharmaceutics14020392/s1, Table S1: Primers forward (F), reverse (R), and probes (P) used for *Eimeria* identification, Figure S1: Small intestine stained with Alcian Blue and PAS, Figure S2: Light intensity graph obtained from particle tracking analysis, Figure S3: Photomicrographs obtained from the NCS100t formulation after 60 days of storage under refrigeration and keeping the formulation at rest, Table S2: Physicochemical parameters of nanoformulations containing toltrazuril after exposure to simulated gastric and intestinal media, Table S3: Nanotechnological properties of formulations after dilution in water, Figure S4: Copy number/PCR of *Eimeria* spp. in cage excreta through days 17 to 21 of the experiment, Figure S5: Plot of median Likert score vs. group.
