# Supplementary material for: Toltrazuril-Loaded Polymeric Nanocapsules as a Promising Approach for the Preventive Control of Coccidiosis in Poultry
Source: Pharmaceutics. 2022 Feb 10;14(2):392. doi: 10.3390/pharmaceutics14020392 (PMC8878044; doi:10.3390/pharmaceutics14020392)
Supplement: Supplementary file 1 [file pharmaceutics-14-00392-s001.zip › pharmaceutics-1534588-supplementary.pdf]

pharmaceutics

MDPI

Supplementary Materials: Toltrazuril-Loaded Polymeric Nanocapsules as a Promising Approach for the Preventive Control of Coccidiosis in Poultry

Lana Flávia Baron, Francisco Noé da Fonseca, Shaiana Salete Maciag, Franciana Aparecida Volpato Bellaver, Adriana Mércia Guaratini Ibeli, Marcos Antônio Zanella Mores, Gabryelle Furtado de Almeida, Silvia Stanisçuaski Guterres, Ana Paula Almeida Bastos, Karina Paese

Table S1. Primers forward (F), reverse (R) and probes (P) used for *Eimeria* identification.

Species	Sequence source	Primer and probe sequences	Reference
E. acervulina	Ac-AD18-953	F: GCAGTCCGATGAAAGGTATTTG<br>R: GAAGCGAAATGTTAGGCCATCT<br>P: [6FAM] ACAGTCCCCGCTGATGGTGTAACG[BHQ1]	15
E. maxima	EmMIC1	F: TCGTTGCATTCGACAGATTC<br>R: TAGCGACTGCTCAAGGGTTT<br>P: [6FAM] ATTGTCCAGCCAAGGTTCCCTTCG[BHQ1]	15
E. tenella	Tn-E03-1161	F: TCGTCTTTGGCTGGCTATTC<br>R: CAGAGAGTCGCCGTCACAGT<br>P: [6FAM] CTGGAAAGCGTCTCCTTCAATGCG[BHQ1]	15
E. spp	5S rRNA	F: TCATCACCCAAAGGGATT<br>R: TTCATACTGCGTCTAATGCAC<br>P: [6FAM] CGCCGCTTAACTTCGGAGTTCAGATGGGAT [BHQ1]	16

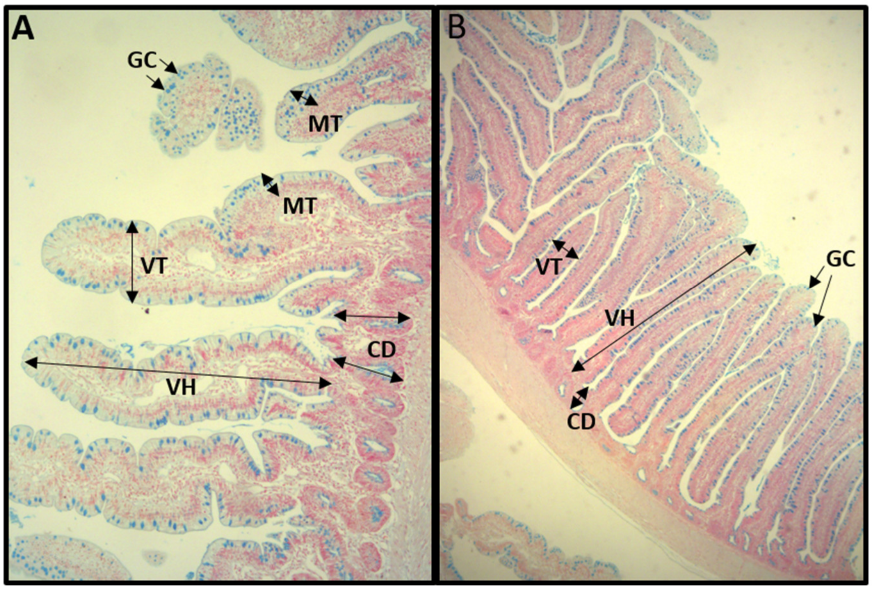

Figure S1. Small intestine stained with Alcian Blue & PAS. Cells stained in blue are goblet cells (GC). VH: villus height; CD: crypt depth; VT: villus thickness; MT: mucosal thickness. Magnification, 200× (**A**) and 100× (**B**).

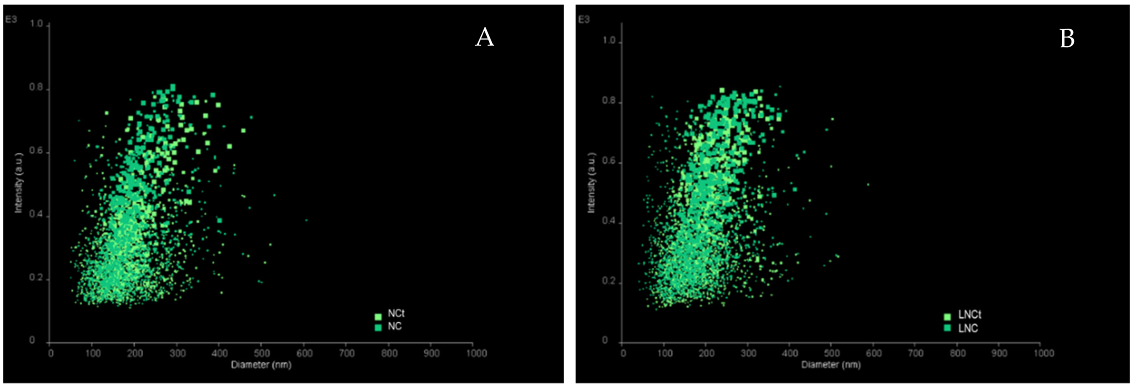

Figure S2. Light intensity graph obtained from particle tracking analysis. (**A**) Eudragit® S100 nanocapsules with toltrazuril ($NC_{S100}t$) or not (NC_{S100}); (**B**) Lipid core nanocapsules with toltrazuril (LNCt) or not (LNC).

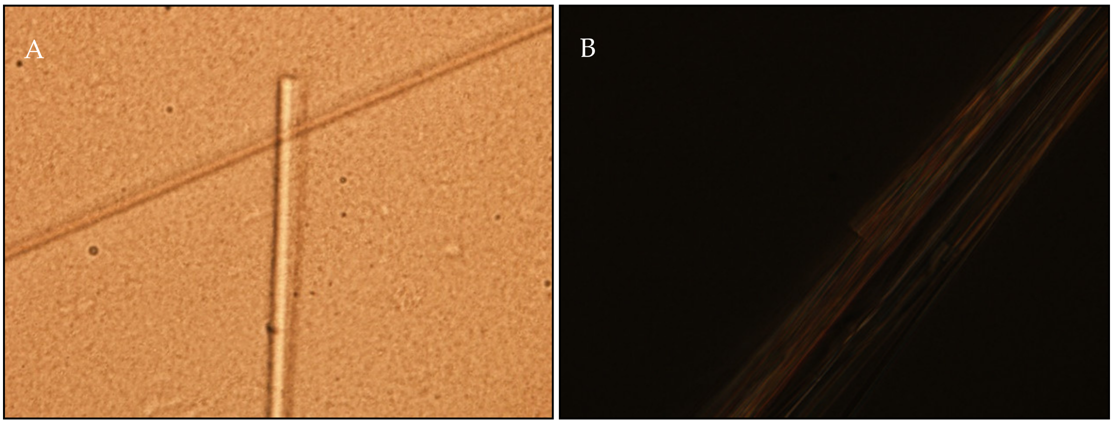

Figure S3. Photomicrographs obtained from the $NC_{S100}t$ formulation after 60 days of storage under refrigeration and keeping the formulation at rest. (**A**) normal light, (**B**) polarized light filter.

Table S2. Physicochemical parameters of nanoformulations containing toltrazuril after exposure to simulated gastric and intestinal media.

**Parameter**		**LNC**	**NC_{S100}**	**LNCt**	**$NC_{S100}t$**
	Before	207 ± 1	154 ± 1	181 ± 2	151 ± 2
z-average (nm)	Gastric step	211 ± 3	152 ± 1	178 ± 1	151 ± 2
	Intestinal step	207 ± 9	159 ± 1	176 ± 2	154 ± 3
	Before	0.145 ± 0.012	0.133 ± 0.013	0.091 ± 0.010	0.116 ± 0.018
PDI	Gastric step	0.171 ± 0.036	0.157 ± 0.021	0.100 ± 0.013	0.122 ± 0.006
	Intestinal step	0.145 ± 0.044	0.140 ± 0.063	0.095 ± 0.008	0.097 ± 0.009
	Before	n.d.	n.d.	100*	100*
Content (%)	Gastric step	n.d.	n.d.	105.24 ± 7.94	92.20 ± 2.23
	Intestinal step	n.d.	n.d.	99.61 ± 2.41	89.98 ±1.58

Values represent mean ± SD (n=3). LNC = blank lipid core nanocapsules; NC_{S100} = blank Eudragit® S100 nanocapsules; LNCt = toltrazuril-loaded lipid core nanocapsules; $NC_{S100}t$ = toltrazuril-loaded Eudragit® S100 nanocapsules formulations; n.d. = not determined. *toltrazuril concentration before exposure to gastric and intestinal media was considered 100%.

Table S3. Nanotechnological properties of formulations after dilution in water.

		LNCt		LNC	
		0 h	24 h	0 h	24 h
	1 (1:10.5)	180.2 ± 4.3	179.3 ± 1.9	210.7 ± 2.5	207.7 ± 2.1
Particle diameter (nm)	2 (1:21)	180.1 ± 2.6	179.0 ± 0.6	-	-
	3 (1:42)	177.9 ± 1.2	179.0 ± 1.8	-	-
Drug content (%)	1 (1:10.5)	109.00 ± 5.18	-	-	-
	2 (1:21)	106.31 ± 6.76	-	-	-

	3 (1:42)	103.71 ± 10.50	-	-	-
Association efficiency (%)	1 (1:10.5)	99.88 ± 0.07	99.93 ± 0.01	-	-
	2 (1:21)	99.80 ± 0.08	99.86 ± 0.03	-	-
	3 (1:42)	99.26 ± 0.91	99.74 ± 0.03	-	-

Values represent mean ± SD (n=3). LNC = blank lipid core nanocapsules; LNCt = toltrazuril-loaded lipid core nanocapsules.

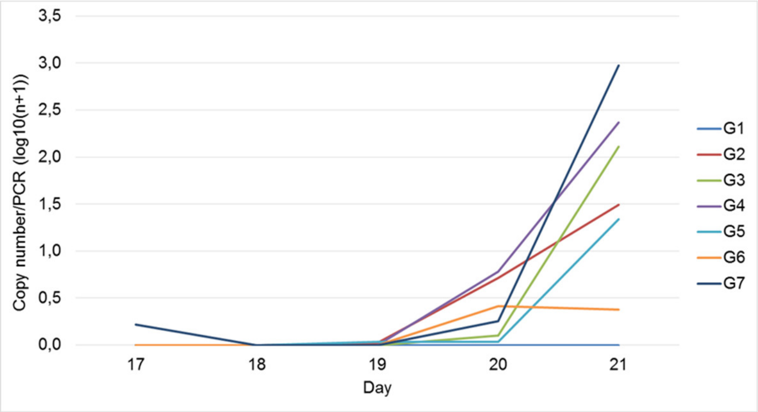

Figure S4. Copy number/PCR of *Eimeria spp*. in cage excreta through days 17 to 21 of the experiment. G1 = control (−); G2 = control (+); G3 = Baycox® (7 mg/kg/day); G4 = LNCt (7 mg/kg/day); G5 = LNCt (3.5 mg/kg/day); G6 = LNCt (1.75 mg/kg/day); G7 = LNC (volume equivalent to G4).

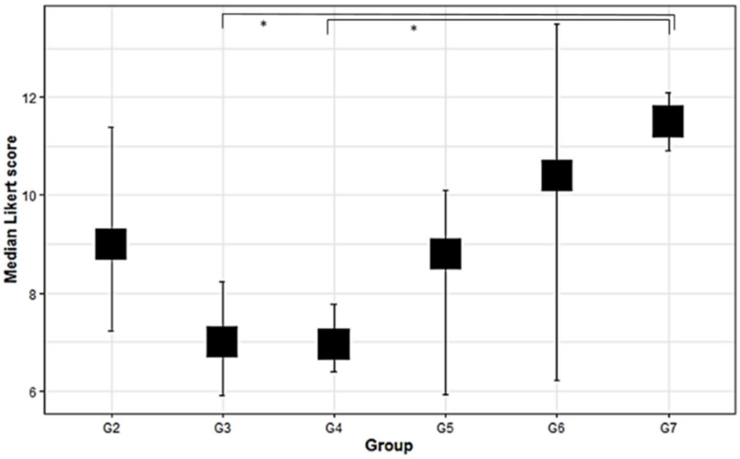

Figure S5. Plot of median Likert score versus group. Error bars indicate the 95% confidence intervals for the median with the percentile method. *Adjusted p-value <0.05. G2 = control (+); G3 = Baycox® (7 mg/kg/day); G4 = LNCt (7 mg/kg/day); G5 = LNCt (3.5 mg/kg/day); G6 = LNCt (1.75 mg/kg/day); G7 = LNC (volume equivalent to G4).
